# PReS-FINAL-2264: Three middle fingers width correlates with maximum mouth opening and is a reliable parameter to identify joint hypermobility in schoolchildren

**DOI:** 10.1186/1546-0096-11-S2-P254

**Published:** 2013-12-05

**Authors:** F Sperotto, G La Falce, F Vittadello, M Balzarin, L Favero, F Zulian

**Affiliations:** 1 Department of Pediatrics, University of Padua, Padua, Italy; 2 Gnatology Unit, Department of Dentistry, University of Padua, Padua, Italy

## Introduction

Maximum mouth opening (MMO) is a useful parameter to identify common temporomandibular joint (TMJ) disorders. Up to now, a few studies addressed the issue on MMO normal values in pediatric population, according to age and/or presence of generalized joint hypermobility (GJH), therefore it is difficult to use it in general medical practice.

## Objectives

Aim of the study was to evaluate the MMO in a cohort of healthy schoolchildren and to propose a new parameter, the Three Middle Fingers Width (TMFW), the distance between 2^nd ^and 4^th ^fingers of the right hand at the level of the lowest nail bed, to evaluate the TMJ hypermobility in children. We also analyzed the relationship between GJH and TMJ hypermobility.

## Methods

We conducted a cross sectional study in a cohort of healthy schoolchildren, aged 8-13 years, by collecting information on family history of GJH and on history of TMJ involvement and performing a physical examination. This included height, weight, body surface area (BSA), body mass index (BMI), and musculoskeletal examination focused on the presence of GJH according to the Beighton criteria (BS≥4/9). TMJ evaluation included a complete gnathological visit, aimed to investigate the presence of TMJ disorders and to evaluate the MMO. The evaluation of TMFW was also performed and the Mouth Opening Ratio (MOR) was consequently calculated by the formula (MMO-TMFW)/MMOx100, adopting a 10% cut-off value to define the TMJ hypermobility.

## Results

Two hundred and eighty-eight schoolchildren, 143 females and 145 males, entered the study. Mean MMO was 45.57 mm (± 5.12) for males and 44.87 mm (± 4.98) for females. Mean TMFW was 43.03 mm (± 4.09) for males and 41.71 mm (± 3.84) for females. Both MMO and TMFW correlate with growth parameters as height, weight, BMI and BSA. 89 (30.9%) subjects showed TMJ hypermobility (MOR>10%). In these subjects and in those with normal MOR MMO correlates with TMFW (r = 0.761, p < 0.001 and r = 0.786, p < 0.001 respectively); The prevalence of subjects with GJH was significantly (p < 0.001) higher in the group with TMJ hypermobility than in the other (44.8% vs 21.5%).

## Conclusion

TMFW correlates with MMO in schoolchildren and may represent a simple and reliable method to evaluate TMJ abnormalities. MOR, as an index to identify TMJ hypermobility, correlates with the presence of GJH and could be included, as an adjunctive point, to the Beighton Criteria.

## Disclosure of interest

None declared.

